# The Alcatraz‐Strategy: a roadmap to break the connectivity barrier in malignant brain tumours

**DOI:** 10.1002/1878-0261.13642

**Published:** 2024-04-03

**Authors:** Matthias Schneider, Anna‐Laura Potthoff, Georg Karpel‐Massler, Patrick Schuss, Markus D. Siegelin, Klaus‐Michael Debatin, Hugues Duffau, Hartmut Vatter, Ulrich Herrlinger, Mike‐Andrew Westhoff

**Affiliations:** ^1^ Department of Neurosurgery University Hospital Bonn Germany; ^2^ Brain Tumour Translational Research Group University Hospital Bonn Germany; ^3^ Department of Neurosurgery University Medical Center Ulm Germany; ^4^ Department of Neurosurgery BG Klinikum Unfallkrankenhaus Berlin gGmbH Germany; ^5^ Department of Pathology and Cell Biology Columbia University Irving Medical Center New York NY USA; ^6^ Department of Pediatrics and Adolescent Medicine University Medical Center Ulm Germany; ^7^ Department of Neurosurgery, Gui de Chauliac Hospital Montpellier University Medical Center France; ^8^ Team “Plasticity of Central Nervous System, Stem Cells and Glial Tumors,” National Institute for Health and Medical Research (INSERM), U1191 Laboratory, Institute of Functional Genomics University of Montpellier France; ^9^ Division of Clinical Neuro‐Oncology, Department of Neurology University Hospital Bonn Germany

**Keywords:** Alcatraz, brain tumors, gap junctions, supramarginal resection, tumour microtubes, tumour networks

## Abstract

In recent years, the discovery of functional and communicative cellular tumour networks has led to a new understanding of malignant primary brain tumours. In this review, the authors shed light on the diverse nature of cell‐to‐cell connections in brain tumours and propose an innovative treatment approach to address the detrimental connectivity of these networks. The proposed therapeutic outlook revolves around three main strategies: (a) supramarginal resection removing a substantial portion of the communicating tumour cell front far beyond the gadolinium‐enhancing tumour mass, (b) morphological isolation at the single cell level disrupting structural cell‐to‐cell contacts facilitated by elongated cellular membrane protrusions known as tumour microtubes (TMs), and (c) functional isolation at the single cell level blocking TM‐mediated intercellular cytosolic exchange and inhibiting neuronal excitatory input into the malignant network. We draw an analogy between the proposed therapeutic outlook and the Alcatraz Federal Penitentiary, where inmates faced an impassable sea barrier and experienced both spatial and functional isolation within individual cells. Based on current translational efforts and ongoing clinical trials, we propose the Alcatraz‐Strategy as a promising framework to tackle the harmful effects of cellular brain tumour networks.

## Graphical abstract

This review introduces the ‘Alcatraz‐Strategy’ for malignant brain tumours, drawing parallels to Alcatraz's isolating barriers. By disrupting cellular tumour networks through supramarginal resection and targeted pharmacological therapy, it aims to decouple tumour cells, akin to the prison's spatial and functional inmate isolation. This approach offers a promising framework to counteract the malignant connectivity of brain tumour networks.

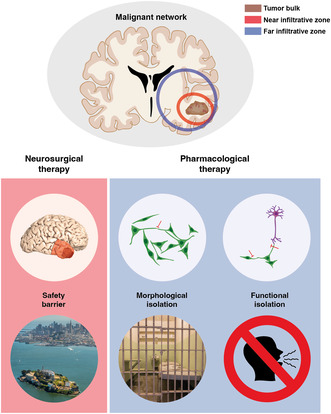

**Abbreviations**
AC/MES: astrocyte‐ and mesenchymal‐like cellular states
AMPAR: α‐amino‐3‐hydroxy‐5‐methyl‐4‐isoxazolepropionic acid receptors
ATL: anterior temporal lobectomy
ATP: adenosine triphosphate
BDNF: brain‐derived neurotrophic factor
cGAMP: cyclic guanosine monophosphate‐adenosine monophosphate
CNS: central nervous system
Cx43: connexin‐43
EudraCT: European Union Drug regulating Authorities Clinical Trials Database (EudraCT)
FDA: Food and Drug Administration
FLAIR: fluid‐attenuated inversion recovery
Gap43: growth‐associated protein 43
ICWs: intracellular calcium waves
IDH: isocitrate dehydrogenase
IFNα: interferon‐α
IP3: inositol triphosphate
MFA: meclofenamate
MGMT: O6‐methylguanine‐DNA methyltransferase
MRI: magnetic resonance imaging
NF‐κB: nuclear factor kappa B
NGF: nerve growth factor
NLGN‐3: Neuroligin 3
NMDA receptor: *N*‐methyl‐d‐aspartate receptor
NPC: neural progenitor cell
NT‐4: neurotrophin‐4
OPC: oligodendrocyte precursor cell
PI3K: phosphatidylinositol 3‐kinase
RT: radiation therapy
STAT1: signal transducer and activator of transcription 1
STING: stimulator of interferon genes
TM: tumour microtube
TMZ: temozolomide
TNF: tumour necrosis factor
Ttyh1: tweety homologue
WHO: World Health Organization

## 1 Introduction

Gliomas are the most common type of primary brain tumours [1]. Among them, glioblastoma (referred to as glioblastoma CNS WHO grade 4, isocitrate dehydrogenase (IDH) wildtype [2]) stands out as the most prevalent with an incidence rate of about 3/100 000 [1]. The particularly aggressive nature of glioblastoma is underscored by a median overall survival rate of merely 15–18 months [3], classifying it as an incurable disease up until now.

Despite considerable efforts in research and therapeutic advancements, a definitive cure remains elusive. The persistent therapeutic failures associated with these tumours can be attributed to several inherent characteristics. Primarily, their almost unlimited proliferative capacity allows for rapid growth and expansion [4]. This is further exacerbated by their microinvasive nature, enabling the tumour to extensively infiltrate the surrounding brain tissue [5]. Additionally, the presence of intratumour heterogeneity raises the likelihood of certain tumour cells surviving therapy‐induced elimination [6]. Ongoing diversification of tumour cell phenotypes during treatment empowers them to adapt to the selective pressures imposed by therapy, facilitating the development of *de novo* resistance and tumour relapse [7].

Recent advances in tumour biology have led to a refined understanding of brain tumours, moving away from the simplistic view of unregulated cellular proliferation. Current research has shown that tumours can be envisioned as cellular networks, with intricate interactions and communication pathways, reminiscent to functional organs [8]. Within these cellular interactions in tumour networks, two primary dimensions have been discerned: homotypical interactions, which refer to interactions exclusively between individual tumour cells, and heterotypical interactions that occur between the tumour cells and various other cell entities [8]. Among these heterotypical interactions, neurons have been observed to integrate the tumour into functional neuronal circuits.

In light of this evolved understanding, the present perspective aims to elucidate the foundational principles governing cellular tumour networks. Based on these principles and contemporary first clinical trials – both surgical and medicinal product studies, the authors further conceive a multifaceted treatment approach specifically designed to counteract the multicellular network connectivity in malignant brain tumours.

## 2 Cellular interactions in malignant brain tumour networks

### 2.1 Homotypical tumour cell‐tumour cell interactions

**Fig. 1:**
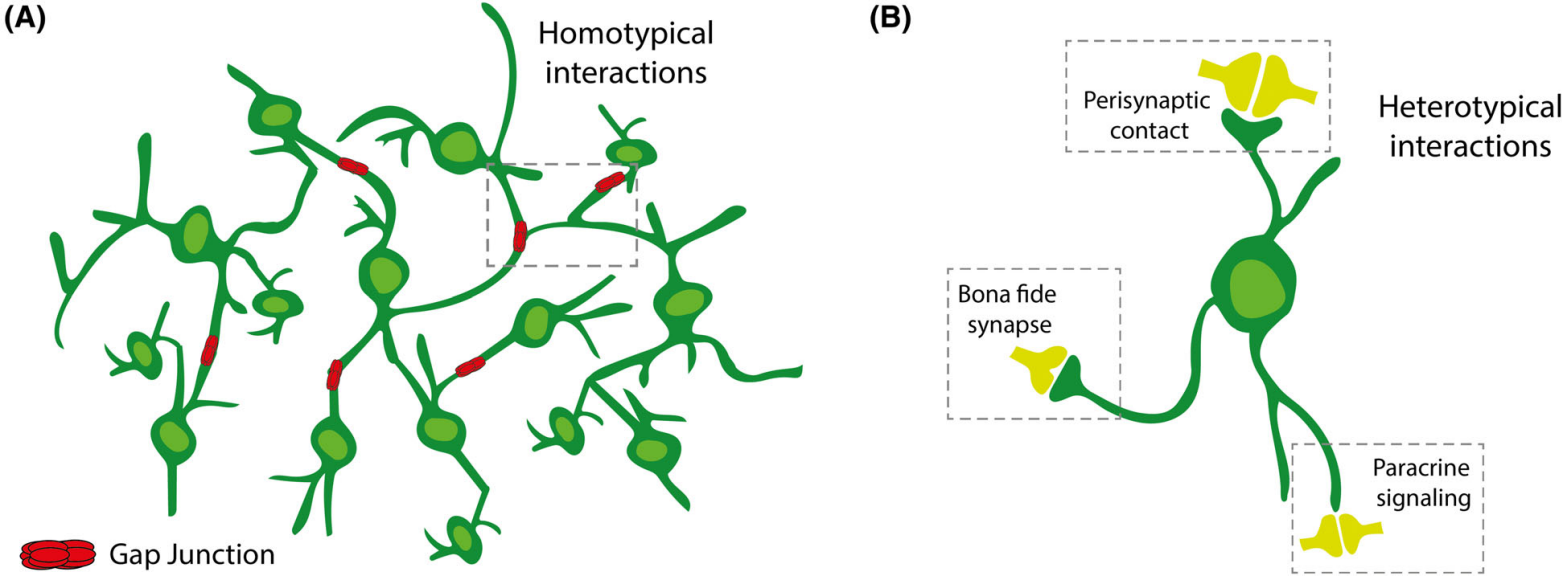
Cellular interactions in brain tumour networks. (A) Homotypical tumour cell‐tumour cell interactions build on ultra‐long membrane protrusions known as TMs. These connections are reinforced by Cx43‐based gap junctions, enabling intercellular communication through cytosolic exchange and bidirectional propagation of intercellular calcium waves. (B) Heterotypical network interactions encompass both direct and indirect communication pathways. Neurogliomal synapses represent direct cell‐to‐cell connections formed between neurons in the presynaptic role and tumour cells in the postsynaptic role. Located alongside TMs, these synapses facilitate the integration of tumour cells into neuronal circuits. Tumour cells can also assume an indirect perisynaptic position. Glutamate released in the synaptic cleft spills over and binds to glutamate receptors on tumour cell surfaces. Heterotypical network interactions include paracrine signaling, where physiological neuronal activity leads to the release of neurotransmitters that influence malignant synaptogenesis. Cx43, connexin‐43; TMs, tumour microtubes.

Within the realm of malignant brain tumour networks, an essential aspect lies in the interactions between individual tumour cells (Fig. 1A). These interactions are facilitated by key cellular components known as tumour microtubes (TMs). TMs are ultralong membrane protrusions, measuring approximately 1.7 μm in width on average, with some extending beyond 500 μm in length [8, 9].

One of the notable features of TMs is their enrichment in contractile forces like myosin IIa, actins and microtubules [9]. These contractile forces play a crucial role in mediating the microinvasion capacity of glioma cells [9]: TMs extend into the surrounding brain and tumour tissue, where they scan the tumour microenvironment and facilitate the formation of new cellular connections [10, 11].

Two morphologically, molecularly and functionally different subtypes of TMs are observed: Tumour cells with non‐connecting TMs are dynamic structures located at the invasive front of tumour cells, reminiscent of axonal growth cones and neurite outgrowths during neurodevelopment [8]. These TMs exhibit enrichment for oligodendrocyte precursor cell (OPC) and neural progenitor cell (NPC)‐like cellular states, as well as neurodevelopmental signatures [12, 13]. Functionally, non‐connecting TMs are associated with behaviours akin to neuronal migration during neurodevelopment, including locomotion, branching migration, and translocation [14]. Tumour cells accompanied by non‐connecting TMs demonstrate increased sensitivity to alkylating chemotherapy [10]. Connecting TMs, conversely, are predominantly situated within the tumour bulk and are responsible for establishing homotypical connections between tumour cells [8]. These TMs exhibit an enrichment of astrocyte‐ and mesenchymal‐like cellular states (AC/MES), along with injury response signatures [12, 13, 15].

Approximately 50% of all tumour cells in glioblastoma become integrated into the TM‐connected homotypical network, conferring heightened resistance to alkylating chemotherapy [10]. A recent study has shown that upon colonizing new brain regions, invasive TM‐non‐connected cells undergo a phenotypic transformation into TM‐connected, slower‐cycling tumor cells, thereby becoming part of the ‘wired‐in’ fraction [16].

Four molecular key drivers of TMs have been discovered: Growth‐associated protein 43 (Gap43) [9], connexin‐43 (Cx43) [9], tweety homologue (Ttyh1) [17] and axon guidance molecules [15]. Gap43, also referred to as neuromodulin, is a cytosolic protein that exhibits high expression levels in axonal growth cones during neurite outgrowth, where it plays a pivotal role in regulating neurite formation during neurodevelopment and aiding in regenerative axon growth processes [18, 19]. In the context of TMs, Gap43 takes on a crucial role by displaying enriched expression at the tips of these ultralong membrane protrusions [9]. This enrichment of Gap43 is instrumental in driving the outgrowth of TMs and facilitating their invasive capabilities into the brain tissue. In this context, Gap43 knockdown in murine glioblastoma models has been observed to inhibit glioma cell invasion based on non‐connecting TMs [10]. Moreover, the inhibition of Gap43 disrupts the formation of intercellular connections between tumour cells mediated by TMs [10], indicating that Gap43 is crucial for establishing homotypical tumour cell–tumour cell connections through TMs. Gap43, within the mature central nervous system, appears to be predominantly involved in long‐term potentiation [20] and memory storage processes [21]. These functions, distinct from its role in neurodevelopment, suggest a potential therapeutic window for targeting Gap43 in adult patients at least for a defined period of time [8].

Cx43 constitutes a further molecular key driver of TMs [9]. Cx43‐based gap junctions are primarily located at the end of connecting TMs and play a vital role in supporting the formation of homotypical connections between tumour cells via TMs [9]. Cx43‐based intercellular gap junctions offer a conduit for the exchange of various small molecules, including calcium ions, adenosine triphosphate (ATP), inositol triphosphate (IP3) and microRNA [9, 22] and facilitate the redistribution of cell organelles, such as mitochondria and cell nuclei [9, 23]. Furthermore, these gap junctions enable the bidirectional spread of intracellular calcium waves (ICWs) [9]. Notably, ICWs are not uniformly distributed within an established network. Instead, they give rise to distinct ‘activity hubs’ characterized by high functional connectivity based on ICWs, alongside areas with lower functional connectivity based on ICWs [15]. These ‘activity hubs’ are inhabited by a population of highly active glioblastoma cells that exhibit autonomous rhythmic calcium oscillations [24]. Selective ablation of these ‘pacemaker’ cells has been shown to compromise global network communication, underscoring the significance of Cx43‐based gap junctions in coordinating tumour cell interactions within malignant brain tumour networks [24]. The expression of Cx43 is influenced by the genes of neurotrophic factors, specifically nerve growth factor (NGF) and neurotrophin‐4 (NT‐4) [25, 26]. These factors are located on both chromosomal parts 1p and 19q [9, 27]. Interestingly, as raised by Osswald et al. [9], 1p/19q co‐deleted tumours may be associated with TM‐poor tumour networks. In contrast, 1p/19q non‐co‐deleted gliomas tend to have more and longer TMs [9]. This distinction might offer a potential explanation for the improved prognosis and particular sensitivity to chemotherapy of 1p/19q co‐deleted oligodendrogliomas. In both human *in vitro* and murine *in vivo* models, knockdown of Cx43 was associated with a reduction in tumour size and a decrease in the number of tumour cells connected via TMs in homotypical tumour cell‐tumour cell connections [9]. Further morphological human tissue sample analysis confirmed that astrocytomas exhibit a greater number and length of TMs compared to oligodendrogliomas and that higher‐grade astrocytomas possess more and longer TMs than their lower‐grade counterparts [9].

Ttyh1 is a calcium‐regulated chloride channel [28] that has been identified as a further molecular key driver of TMs [17]. It exhibits high expression in the membrane of axonal growth cones during neuritogenesis [29]. Notably, Ttyh1 is prominently expressed in the growth cones of invasive non‐connecting TMs and plays a crucial role in regulating TM outgrowth and facilitating malignant cell invasion into the brain [17]. In studies involving knockdown of Ttyh1, a substantial reduction in the number of microinvasive glioblastoma cells was observed, underscoring the importance of Ttyh1 in promoting invasive behaviour [17]. Interestingly, this knockdown of Ttyh1 did not significantly impact TM‐connected tumour cells, highlighting a specific role for Ttyh1 in non‐connecting TMs and their invasive properties [17].

Axon guidance molecules, including NETRIN‐1 and SEMA3A, typically are involved in tissue development, branching morphogenesis and the evolution of three‐dimensional structures during organogenesis [30, 31]. In a preclinical glioblastoma *in‐vitro* model, it has been observed that downregulation of axon guidance signaling pathways is accompanied by a significant reduction in the length of TMs [15]. This reduction in TM length consequently leads to decreased network connectivity [15] underscoring the pivotal role of axon guidance molecules as molecular key drivers of TM‐based network formation.

### 2.2 Heterotypical neuron–tumour cell interactions

Among heterotypical network interactions, interactions between tumour cells and neurons stand out as the most extensively studied. These interactions can emerge as direct and indirect pathways of communication.

Neurogliomal synapses represent bona fide synapses as direct cell‐to‐cell connections that form between neurons in the presynaptic role and tumour cells in the postsynaptic role [32] (Fig. 1B). These synapses are located alongside TMs [33], underscoring another crucial role of TMs – they enable the heterogeneous integration of tumour cells into neuronal circuits. Presynaptic neurons release glutamate triggering excitatory currents in postsynaptic tumour cells through the activation of α‐amino‐3‐hydroxy‐5‐methyl‐4‐isoxazolepropionic acid receptors (AMPAR) and inducing slow inward currents [32, 33]. These electrical events may induce calcium transients within tumour cells, a phenomenon that has been shown to fuel tumour cell proliferation and microinvasion [8, 15, 32, 33].

In addition to direct synaptic connections between neurons and tumour cells, tumour cells can also assume an indirect perisynaptic position (Fig. 1B). In this scenario, glutamate spilled over from the synaptic cleft, binds to glutamate receptors on the tumour cell surface [8, 33]. Such indirect perisynaptic contacts have been identified in the context of breast cancer brain metastases, where breast cancer cells receive glutamatergic signals via *N*‐methyl‐d‐aspartate receptors (NMDAR), thereby promoting malignant cell proliferation [34]. Similarly, morphological perisynaptic interactions between tumour cells and neurons have been described in glioblastoma [33]. However, the presence of NMDAR signaling in glioma lacks clear evidence to date [32, 33], leaving the precise function of perisynaptic interactions in glioma somewhat enigmatic [14].

In the realm of heterotypical network interactions, paracrine signaling unveils yet another dimension of indirect communication (Fig. 1B). It stems from the physiological neuronal activity via the paracrine release of neurotransmitters but influences malignant synaptogenesis. Neuroligin 3 (NLGN‐3), a synaptic protein, is produced by neurons [35]. Following cleavage by the metalloproteinase ADAM10, NLGN‐3 undergoes a transformation into a soluble form, allowing it to engage with glioma cells [36, 37]. This interaction sets off a cascade of events within the glioma cells, culminating in the activation of the PI3K‐mTOR signaling pathway. Brain‐derived neurotrophic factor (BDNF) provides another example; it orchestrates the trafficking of AMPAR to the postsynaptic membrane of glioma cells, a process intricately regulated by neuronal activity (Fig. 1B). These paracrine signals, arising from physiological neuronal activity, drive malignant synaptic plasticity and contribute to tumour progression [38].

## 3 Tumour networks and their role for therapy resistance

Tumour networks play a significant role in therapy resistance, presenting formidable challenges for treatment efficacy. Within this context, four critical aspects shed light on how these networks promote resistance.

### 3.1 Wound healing response

**Fig. 2:**
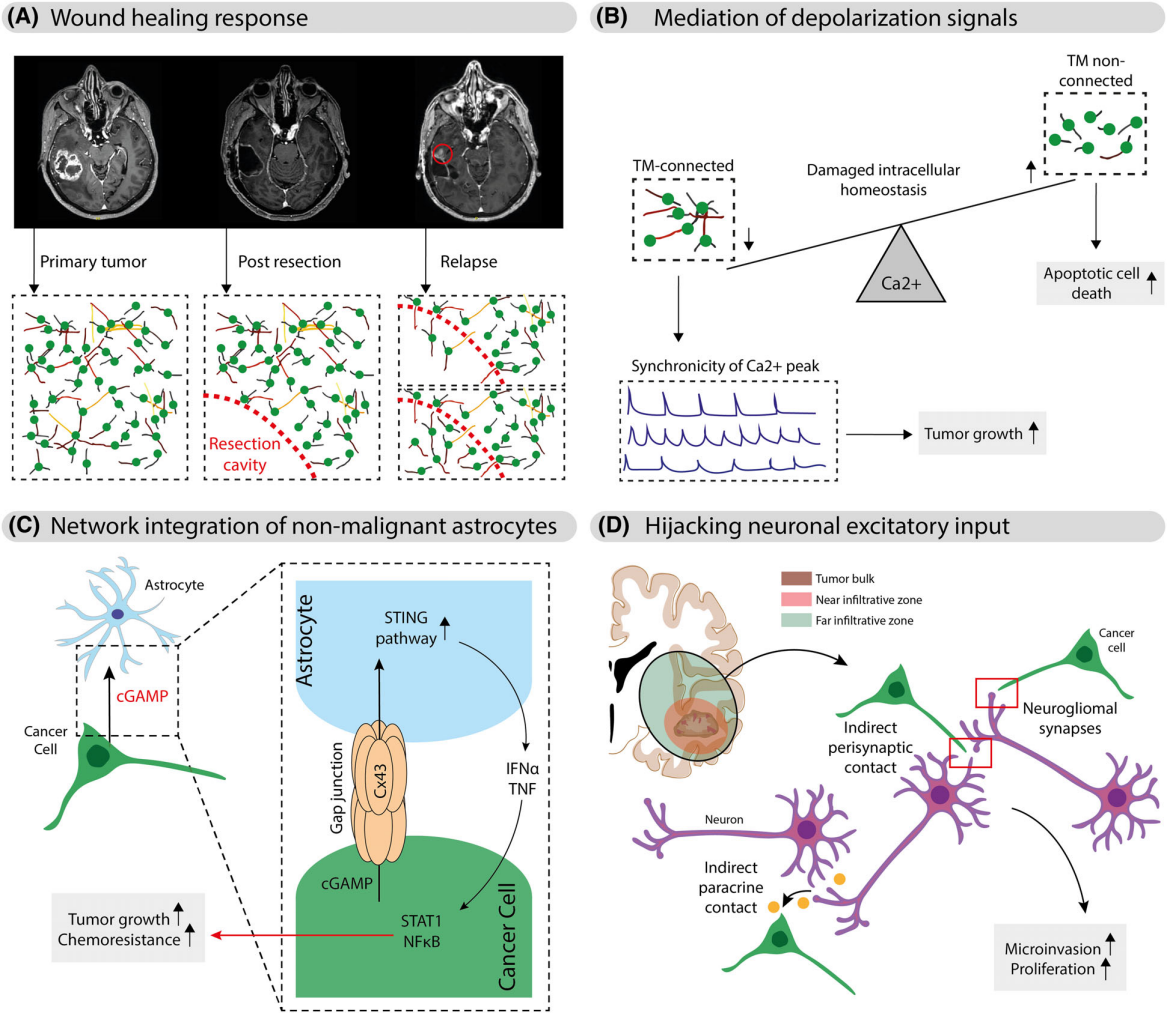
Tumour networks and their mechanisms of resistance. Tumour networks underpin therapy resistance, comprising four key elements. (A) The wound healing response illustrates tumour recurrence near surgical resection sites. Residual tumour cells extend TMs from resection margins, forming connections and facilitating tumour regrowth. These TMs facilitate the formation of new connections and support the migration and proliferation of glioblastoma cells, mirroring the regenerative process of wound healing and contributing to tumour regrowth. (B) Tumour networks exhibit synchronized calcium fluctuations, particularly in TM‐connected cells. Higher calcium concentrations in TM‐non‐connected cells following irradiation are associated with cell death, while TM‐connected cells maintain lower intracellular calcium levels. This synchronization in TM‐connected networks may serve as a protective mechanism, potentially mitigating the adverse effects of toxic metabolites, including calcium and chemotherapeutic drugs, on individual network cells. (C) Heterotypic Cx43‐based gap junctions form between non‐malignant astrocytes and cancer cells, enabling the transfer of cGAMP. This transfer activates the STING pathway in astrocytes. As a result, astrocytes produce inflammatory cytokines like IFNα and TNF, creating a paracrine signaling cascade. These cytokines subsequently activate the STAT1 and NF‐κB pathways in cancer cells driving tumour growth and chemoresistance. (D) Tumour cells integrate into neuronal circuits through direct neurogliomal synapses, indirect perisynaptic contacts and indirect paracrine signaling. Excitatory neuronal input into the malignant network influences two critical aspects of tumour behaviour: it enhances TM dynamics, promoting increased microinvasive capacity and stimulates tumour cell proliferation. Notably, this neurogliomal input may be particularly prevalent in the far infiltrative tumour zone, which persists following surgical tumour bulk resection, posing concerns for long‐term therapy success. cGAMP, cyclic guanosine monophosphate‐adenosine monophosphate; Cx43, connexin‐43; IFNα, interferon‐α; NF‐κB, nuclear factor kappa B; STAT1, signal transducer and activator of transcription 1; TMs, tumour microtubes; TNF, tumour necrosis factor.

Following initial resection, glioblastoma predominantly recurs adjacent to the surgical resection cavity [39]. This recurrence has been attributed to the higher cell density at the resection margin, which decreases with distance from the resection cavity [40]. With the emerging understanding of tumour networks, TMs offer a potential cellular explanation for how residual tumour cells at the resection margin contribute to the reformation of the recurrent tumour mass. Using a murine glioblastoma model, Weil et al. [10] observed that after surgical resection, residual tumour cells extend TMs from the resection margin into the surrounding brain tissue. These TMs form new connections and facilitate the migration of more glioblastoma cells into the resection cavity, eventually leading to the reformation of the tumour mass [10] (Fig. 2A). The mechanism driving this TM‐based tumour reformation is reminiscent of the wound healing process. Similar to how biological systems respond to tissue injury by mobilizing cells for repair and regeneration, glioblastoma cells leverage TMs to re‐establish and grow the TM‐connected tumour mass. In response to the ‘wound’ created by surgical resection, TMs function as a biological scaffold, supporting the migration and proliferation of nonTM‐connected glioblastoma cells into the affected area [11]. This analogy extends to the effects observed following radiotherapy. In this context, TMs grow towards areas of cellular damage, acting as conduits for transmitting cellular components, including cell nuclei, via gap junctions [9]. This process enables self‐repairment of damaged tumour cells within the network, further enhancing the resilience of the malignant connectivity.

### 3.2 Mediation of depolarization signals

Another intriguing facet of tumour networks is their role in mediating depolarization signals. Subtle fluctuations in intracellular calcium levels can disrupt intracellular homeostasis and trigger apoptotic cell death [41]. The synchronicity of calcium peaks in TM‐connected tumour cells, where pacemaker cells set the pace, is notably superior to that observed in TM‐non‐connected tumour cells [9, 24]. High calcium concentrations have been associated with cell death in TM‐non‐connected tumour cells following irradiation, while TM‐connected tumour cells exhibit lower intracellular calcium concentrations [9]. This synchronization within the TM‐connected network may serve as a protective mechanism, potentially buffering local increases in toxic metabolites such as calcium and chemotherapeutic drugs. The distribution of these metabolites via gap junctions between TM‐connected tumour cells could minimize their detrimental effects on individual cells within the network (Fig. 2B).

### 3.3 Network integration of neighbouring non‐malignant astrocytes

Another crucial aspect contributing to therapy resistance is the integration of neighbouring non‐malignant astrocytes into the network. Studies have revealed the formation of heterotypic gap junctions between non‐malignant astrocytes and brain metastatic cells originating from breast and lung cancer. These metastatic cells transfer the second messenger cGAMP to adjacent astrocytes via gap junctions, a process that sets off a chain reaction of events [42]. This transfer of cGAMP activates the STING pathway within astrocytes, leading to the production of inflammatory cytokines, including interferon‐α (IFNα) and tumour necrosis factor (TNF), as part of a paracrine signaling cascade. These factors, acting as paracrine signals, subsequently activate the signal transducer and activator of transcription 1 (STAT1) and nuclear factor kappa B (NF‐κB) pathways in the brain metastatic cells [42]. The activation of these pathways, orchestrated by neighbouring non‐malignant astrocytes, plays a pivotal role in bolstering tumour growth and enhancing chemoresistance in brain metastatic cells [42].

### 3.4 Hijacking neuronal excitatory input

As outlined above, several forms of how tumour cells integrate into neuronal circuits have been described, including direct neurogliomal synapses, indirect perisynaptic contact and indirect paracrine signaling mechanisms. Excitatory neuronal input into the malignant network has been shown to promote two critical aspects of tumour behaviour: (a) The study of Venkataramani et al. [33] shows that excitatory neuronal input fosters TM dynamics within the tumour network. This heightened TM activity culminates in an enhanced microinvasive capacity of tumour cells. TMs play a vital role in facilitating the infiltration of glioblastoma cells into the surrounding brain tissue, thereby contributing to tumour progression [33]. Specifically, TM‐unconnected glioblastoma cells in the infiltrative tumour zones display an AMPA‐receptor phenotype distinct from that in the tumour core, resembling that of neural progenitor cells and rendering these cells particularly responsive to the proliferative effects of glutamate [11, 33]. (b) Excitatory neuronal input leads to increased tumour cell proliferation [33]. It is essential to recognize that this neurogliomal input may be particularly prevalent within the far infiltrative tumour zone, which often persists postoperatively (Fig. 2D). The persistence of excitatory neuronal input in this tumour zone raises concerns regarding its potential role in long‐term therapy failure. Specifically, it may facilitate the reformation of connected tumour cells following surgery, ultimately giving rise to the reestablishment of a new tumour bulk. Understanding the influence of excitatory neuronal input on the dynamics of the malignant network is crucial for developing effective therapeutic strategies that address these challenges.

## 4 Strategies for network disconnection

The discovery of cellular tumour networks has revolutionized our understanding of malignant brain tumours. They are not merely uncontrolled aggregates of proliferating cells; instead, they constitute complex networks of interconnected cellular components, operating within a hierarchical structure [8]. This revelation has opened up a new dimension in the realm of therapy, one that seeks to dismantle the malignant connectivity existing both at the functional and morphological levels.

To achieve the most effective network disconnection, it becomes imperative to target both homotypical and heterotypical network interactions. This necessitates the development of multifaceted therapeutic approaches, combining advanced surgical techniques with pharmacological interventions. However, the translation of these strategies from theory to clinical practice is a critical step. In the following, we shift our focus towards translational strategies for network disconnection, exploring approaches with the potential for clinical translation or those on the verge of clinical implementation. Crucially, these therapies must establish a therapeutic window that effectively halts brain tumour progression while simultaneously safeguarding the critical connectivity of the CNS, recognizing the fundamental significance of this connectivity for physiological CNS functioning.

The focus of this section on glioblastoma reflects the current stage of clinical research in network‐targeted therapies for primary brain tumours, where initial translational efforts into clinical trials have been exclusively observed in glioblastoma.

### 4.1 Extended resection of network zones

Glioblastoma cells are known to reside at very distant sites from the gadolinium‐enhancing solid tumour bulk [43, 44], with reports of their presence even in the opposite hemisphere [45]. As discussed above these tumour cells do not solely comprise individual microinvasive entities but also possess the capability to establish flexible and interconnected cellular networks within these remote tumour zones. This unique ability is supposed to constitute a contributing factor to long‐term therapy failures. Consequently, there has been a growing recognition of the significance of supramarginal resection, a surgical approach that extends well beyond the boundaries of the enhancing tumour bulk [8, 46]. In contrast to conventional gross‐total resection (GTR), which primarily focuses on eliminating the enhancing tumour mass, supramarginal resection takes a more comprehensive approach. It involves the resection not only of the complete network connectivity within the bulky tumour but also extends to the nearby infiltrative zone and additionally permits partial resection of tumour networks in the distant infiltrative tumour zone. Several studies have indicated that supramarginal resection may confer a substantial advantage in terms of long‐term survival when compared to traditional GTR [8, 46]. This benefit, in the context of new insights into tumour networks, may not be solely due to a reduction in tumour mass. Supramarginal resection may target specific network‐based resistance mechanisms inherent to the tumour's biology, going beyond traditional approaches. Supramarginal resection is effective in completely removing the TM‐connected cell fraction in both the tumour bulk and near infiltrative zone. This may be significant because TM‐connected cells are more resistant to radiochemotherapy, likely due to synchronized calcium signaling (see Section 3.2). By extensively resecting TM‐connected cells, a larger portion of the tumour contributing to treatment resistance via calcium peak synchrony is removed. Moreover, supramarginal resection targets not only the TM‐connected tumour cells within the tumour bulk and near infiltrative zone but also extends to the far infiltrative tumour zone. This is critical, as the prevalence of infiltrative nonTM‐connected tumour cells may decrease with increasing distance from the tumour bulk. By removing parts of the far infiltrative zone, supramarginal resection may weaken the wound healing response (see Section 3.1). In this context, postoperative residual invading tumour cells in the far infiltration zone expected to be predominantly nonTM‐connected extend TMs into the resection cavity, thereby facilitating tumour mass reformation [10]. Hence, removing nonTM‐connected cells through supramarginal resection may impede the process of TM formation and tumour mass reformation postsurgery. However, it is essential to acknowledge that existing scientific investigations into supramarginal resection primarily consist of retrospective case series from individual neurosurgical centres [13, 14]. Furthermore, the comparability of these studies is complicated by discrepancies in their definitions of the extent of supramarginal resection [15]. Consequently, despite individual promising retrospective data, the overall value of supramarginal resection in glioblastoma surgery remains unclear [47].

One critical aspect that comes to the forefront pertains to the definition of the extent of resection within a supramarginal resection regimen. Various approaches have been proposed and described to address this issue. One approach defines the extent of supramarginal resection as the percentage of the preoperative fluid‐attenuated inversion recovery (FLAIR) or T2‐FLAIR volume located beyond the boundaries of the gadolinium‐enhancing tumour area that was resected [48, 49, 50]. An observational study indicated that a resection of at least 20% of the preoperative FLAIR volume beyond the gadolinium‐enhancing tumour area was associated with beneficial overall survival (OS). However, it was observed that resections greater than 60% did not exert a significant influence on OS [50]. In a multicentre Norwegian randomized clinical trial (NCT04243005), the aim of supramarginal resection is to achieve a margin of at least 10 mm, which is considered feasible prior to surgery. This resection is guided by the T2 volume, representing the zone of oedema, where the goal is to remove as much of this zone as possible (or beyond) while ensuring safety. The study commenced in June 2020 and its estimated completion date is June 2030 [51].

Lobectomy represents another facet of supramarginal resection. The key advantage of this method lies in its strict anatomically defined boundaries, allowing for enhanced comparability and precision. This approach has been described for various brain lobes, including the frontal [52, 53], temporal [52, 53, 54] and occipital lobes [53]. A significant development in this domain is the multicentre European randomized phase III ATLAS/NOA‐29‐trial, set to commence in February 2024. This prospective trial aims to translate the concept of anterior temporal lobectomy (ATL) from epilepsy surgery [55] to the field of glioblastoma surgery, particularly for cases involving temporo‐lateral glioblastomas. Retrospective data on supramarginal resection via the ATL are promising, offering several notable benefits. Patients undergoing this approach have exhibited a substantial survival advantage [52, 54] without incurring a negative postoperative risk profile [56]. Additionally, the procedure has shown superior outcomes in terms of seizure status when compared to conventional temporal GTR [57] and data from epilepsy surgery suggest the preservation of overall cognitive performance for the ATL approach [58].

Another avenue on supramarginal resection approaches involves defining the extent of resection according to functional boundaries. This approach utilizes direct electrical stimulation mapping of the cortex and subcortical white matter tracts as well as real‐time cognitive monitoring in awake patients undergoing resections. As a result such an approach offers a unique opportunity to set functional boundaries for supramarginal resection [59, 60, 61]. In contrast to traditional approaches that rely on interindividual non‐varying extents of resection based on strict anatomical landmarks, this resection regime is grounded in the functional connectome of the brain and presents a more personalized and adaptable surgical concept [62, 63]. In terms of a ‘surgery à la carte’ approach, it is set to allow for supramarginal resection even in non‐preselected and traditionally considered eloquent brain areas. Numerous studies indicate the feasibility of such approaches [60, 61, 63, 64, 65] due to mechanisms of neural network reconfiguration induced by glioma progression following surgery [66]. By mapping and monitoring the functional boundaries of the brain in real‐time during awake procedures, surgeons can make precise decisions about the extent of resection, ensuring that critical functional neural circuits are spared while targeting the malignant networks even within the peritumoural zone [67]. Importantly, this surgical strategy also safeguards the intricate cognitive and emotional functions [68, 69] essential for maintaining a high quality of life, allowing 97% of patients to regain their capacity to work [70]. Furthermore, as epilepsy is primarily associated with peripheral infiltration rather than the tumour core in diffuse gliomas, supramarginal resection holds promise for improving seizure control [57, 71]. These findings provide a compelling argument for further exploration and integration of functional boundary‐based supramarginal resection into the clinical management of high‐grade gliomas. In addition to malignant primary brain tumours, the technique of supramarginal resection is garnering increased attention in the treatment of brain metastasis [72]. This shift in focus is partly due to the observed invasion patterns in brain metastases. While these metastases typically show less infiltrative growth compared to malignant astrocytomas, postmortem studies have nonetheless uncovered notable invasion patterns in brain metastases as well [73]. These patterns, involving infiltrating metastatic cells, have been suggested to play a role in decreasing overall survival rates [74]. A retrospective analysis has shown that supramarginal resection, which involves extending the GTR margin by an additional 5 mm, is associated with enhanced 2‐year local tumour control and an increase in overall survival rates [75]. However, it is imperative to acknowledge that the efficacy of surgical interventions in metastatic disease is significantly influenced by the systemic tumour condition [72]. Consistent with this understanding, several retrospective studies have indicated that the benefits of supramarginal resection, particularly in terms of prolonged survival, are primarily observed in patients with controlled extracranial disease [76, 77].

### 4.2 Morphological tumour network destruction

Within a multimodal network‐targeted treatment approach, the concept of morphological network destruction emerges with a clear objective: to achieve the isolation of individual tumour cells interconnected within the postoperatively remaining far infiltration zone. This strategy is firmly grounded in the disruption of TMs, recognizing them as the critical elements underpinning these networks. With regard to a translational direction, two pharmacological agents have emerged as potential candidates for this task: ST‐401 and Meclofenamate (MFA). Agent ST‐401 reversibly reduces microtubule assembly that triggers a mitotic delay and cell death during interphase [78]. This mode of action leads to the inhibition of TM formation [78]. In murine glioblastoma models subjected to *in vivo* testing, ST‐401 has exhibited notable antitumor activity. Moreover, it has demonstrated the potential to enhance the therapeutic efficacy of standard treatments such as temozolomide (TMZ) and radiation therapy (RT) [78]. It is crucial to critically note that the antitumoural effect of ST‐401, stemming from the inhibition of microtubule assembly and the resulting mitotic delay [78], might not solely be attributed to the inhibition of malignant connectivity. One particularly encouraging feature of ST‐401 is its formulation optimized for high blood–brain barrier permeability. Notably, studies conducted in murine models have revealed no significant concerns regarding toxicity [78]. It is imperative to acknowledge, however, that as of the present, ST‐401 has not secured approval from the Food and Drug Administration (FDA). This absence of regulatory approval represents a noteworthy limitation, impeding its timely evaluation in the clinical setting.

Meclofenamate (MFA) stands out as an FDA‐approved drug with an established track record as a nonsteroidal anti‐inflammatory drug. Recent *in vitro* studies have demonstrated that MFA leads to a downregulation of axon guidance molecule signaling pathways in glioblastoma cells, resulting in a significant reduction in the length of TMs [15]. This reduction in TM length is of paramount importance as it contributes to the morphological demolition of the TM‐based network architecture within glioblastomas. At the transcriptional level, MFA has been found to induce reprogramming of developmental cellular profiles. Specifically, it redirects cellular states associated with highly connected tumour cells towards a less connectivity‐proficient state [15]. This observation raises the intriguing possibility of establishing a therapeutic window for MFA, targeting the fraction of highly connected glioblastoma cells while simultaneously exhibiting low side effect profiles in the adult physiological brain, where developmental programmes are limited [8, 79]. Regarding its FDA approval, MFA emerges as the first potential drug targeting TMs to undergo clinical evaluation. The MecMeth/NOA24‐trial, a multicentre Germany‐wide investigator‐initiated trial registered under the European Union Drug Regulating Authorities Clinical Trials database (EudraCT) number 2021‐000708‐39 [80], is spearheading this exploration. In its phase I part, this trial focuses on assessing the safety of a combinatory approach of MFA and TMZ in patients with recurrent O6‐methylguanine‐DNA methyltransferase (MGMT) promotor‐methylated glioblastoma. The trial also contains a subsequent randomized phase II part, where the primary objective is to detect the efficacy of this combinatory approach [80]. A noteworthy feature of this trial is its inclusion of tumour resection after the initiation of MFA and TMZ therapy. This aspect provides the opportunity to investigate intratumoural MFA levels and assess the effects of MFA on TM formation within the complete human *in vivo* setting [80].

### 4.3 Functional tumour network destruction

In the pursuit of inhibiting tumour networks at a functional level, one avenue of interest lies in the inhibition of gap junction‐mediated intercellular communication. This approach hinges on the pivotal role of Cx43 as a molecular key driver of TMs. As such, Cx43‐based gap junction‐mediated intercellular cytosolic exchange emerges as a critical component of cell‐to‐cell communication within these networks, making gap junction inhibitors a focal point of exploration. Several drugs have exhibited the capacity to inhibit gap junctions, including carbenoxolone and INI‐0602 [81, 82, 83]. However, the current lack of FDA approval for these drugs hampers further exploration in the clinical setting. MFA, in addition to its effects on TM morphology, has demonstrated to exert gap junction‐inhibitory effects within glioblastoma networks. Notably, MFA inhibits the intercellular cytosolic traffic of small molecules and cell organelles [15, 84]. Furthermore, it interferes with the bidirectional spread of ICWs, thus affecting the dynamic signaling processes within glioblastoma networks [15]. Recent data have indicated that autonomous rhythmic calcium oscillations in pacemaker cells remain unaffected by MFA. However, the forwarding of these oscillations within the activity hubs or between activity hubs is inhibited [24]. Therefore, in addition to its morphological effects on the malignant network architecture, as discussed above, MFA may also contribute to a ‘functional’ demolition of the TM‐based network connectivity. This dual impact on both structural and functional aspects of the network makes MFA a promising candidate for clinical exploration and the ongoing MecMeth/NOA‐24 trial [80] will shed light on its effects in the human *in vivo* setting. Gap junctions are also the target in an early clinical trial investigating the inhibition of intercellular connectivity in the field of brain metastases (NCT02429570). This study explores the inhibition of intercellular communication through gap junctions between brain metastasis cells and astrocytes using MFA. The aim is to prevent tumour cells from inducing inflammatory cytokines in astrocytes through gap junctions, which in turn promote growth and therapy resistance of tumour cells via a paracrine signaling cascade [42]. Thus, as a central aspect of therapy resistance via tumour networks (see Fig. 2C), the gap junction‐mediated functional coupling between tumour cells and non‐malignant cells has advanced into clinical investigations, encompassing not only primary but also secondary brain tumours.

Another approach to functionally target malignant tumour networks may involve the inhibition of neuronal input into these networks. Specifically, targeting neurogliomal synapse activation, a critical phenomenon in the dynamic interactions, is of interest. Two anti‐epileptic drugs, talampanel and perampanel, have shown potential as non‐competitive AMPAR antagonists. These drugs act by targeting the postsynaptic membrane of tumour cells within neurogliomal bona fide synapses. Talampanel was evaluated in a small phase II trial involving recurrent glioblastoma patients. When administered alongside standard‐of‐care adjuvant treatment, talampanel did not demonstrate significant survival‐prolonging effects [85]. However, a larger multi‐centre phase II trial explored the use of talampanel in newly diagnosed glioblastoma cases. In this study, even though a higher percentage of patients had an unmethylated MGMT promotor status, the talampanel‐treated group exhibited prolonged survival [86]. Despite these promising results, the practical limitation of talampanel lies in its short biological half‐life, which is approximately 3 h [87]. This short duration of action necessitates multiple doses per day, rendering it impractical for clinical development. Consequently, the decision was made to discontinue the clinical development of talampanel. In contrast to talampanel, perampanel presents a more promising option for inhibiting neuronal input into malignant tumour networks. This anti‐epileptic drug has gained FDA approval and possesses a significantly longer half‐life in humans, lasting more than 24 h [88]. Currently, two clinical trials, NCT04497142 and NCT04650204, are investigating the effects of perampanel on peritumoural hyperexcitability and its potential to reduce seizure frequency in patients with high‐grade gliomas. The planned PERSURGE‐trial, registered under NCT04202159, represents a significant step in understanding the potential of perampanel in functional cellular disconnection in recurrent glioblastoma. This window‐of‐opportunity trial will administer perampanel perioperatively as an add‐on antiepileptic drug, following a double‐blind design. The primary outcome measures of the study will include evaluating changes in MRI findings and assessing alterations in molecular and morphological tumour cell connectivity within the resected tumour tissue [8].

In addition to the previously discussed approaches, another method for functionally inhibiting malignant tumour networks involves the inhibition of neuronal paracrine signaling. Specifically, two inhibitors, GI254023X and INCB7839, target the metalloprotease ADAM10, which plays a crucial role in this process. ADAM10 is responsible for cleaving full‐length NLGN3 into extracellular NLGN3, which is subsequently secreted by neurons [35, 36, 37]. Venkatesh et al. [37] have provided evidence that ADAM10 inhibitors can effectively reduce tumour cell proliferation in a murine xenograft glioblastoma model. Of particular interest, INCB7839 is currently undergoing evaluation in a phase I clinical trial (NCT04295759). This trial focuses on its potential in the treatment of recurrent or progressive paediatric high‐grade glioma.

## 5 Perspectives and conclusions

The recent advancements in understanding tumour networks have given rise to new therapeutic strategies poised for translation into clinical practice. These translational efforts and ongoing clinical trials are converging to create a therapeutic scenario that takes aim at the malignant network through a multifaceted approach. In this scenario, supramarginal resection, currently under prospective assessment in two clinical trials (ATLAS/NOA‐29 and NCT04243005), emerges as the surgical approach of choice. Supramarginal resection involves the excision of brain tissue infiltrated by the tumour, extending beyond the gadolinium‐enhancing regions on imaging. The goal of supramarginal resection is to completely remove the network components of the tumour bulk and near infiltrative zone and to significantly reduce parts of the malignant connectivity in the far infiltrative zone. Subsequently, postoperative residual network components in the distant infiltration zone are targeted pharmacologically. This pharmacological approach is designed to prevent both homotypical and heterotypical connections among nonTM‐connected tumour cells and to decouple existing connections in TM‐connected, slower‐cycling cells, achieving this on both the morphological and functional level. On the morphological front, the inhibition of TM formation and outgrowth is exemplified by the use of MFA, as explored in the MecMeth/NOA‐24 trial. Preclinical data indicate that MFA impedes the formation and outgrowth of TMs, resulting in a notable morphological transformation that isolates individual tumour cells from one another. On the functional level, network disconnection is achieved through two key mechanisms: (a) Gap junction inhibition and (b) inhibition of excitatory neuronal input into the malignant network. In the former context, MFA shows promise. Besides its impact on network architecture, preclinical data indicates that MFA impedes intercellular communication by blocking gap junctions. This disruption hinders the exchange of cytosolic molecules between neighbouring tumour cells and additionally inhibits the propagation of ICWs across the network. Within the context of inhibiting excitatory neuronal input into the malignant network, perampanel may take centre stage as currently explored in the PERSURGE‐trial. Perampanel operates as an antagonist of AMPARs at the postsynaptic tumour cell membrane within neurogliomal synapses. Through this inhibition, perampanel may effectively isolate tumour cells from neural interactions, further compromising the functional integrity of the network. In essence, this therapeutic approach is designed to halt the major forms of network formation, effectively isolating individual cells within already established or emerging/expanding malignant networks. This multifaceted disconnection strategy weakens the network's resilience and prepares the isolated cells to become more susceptible to the cytotoxic effects of chemotherapy.

**Fig. 3:**
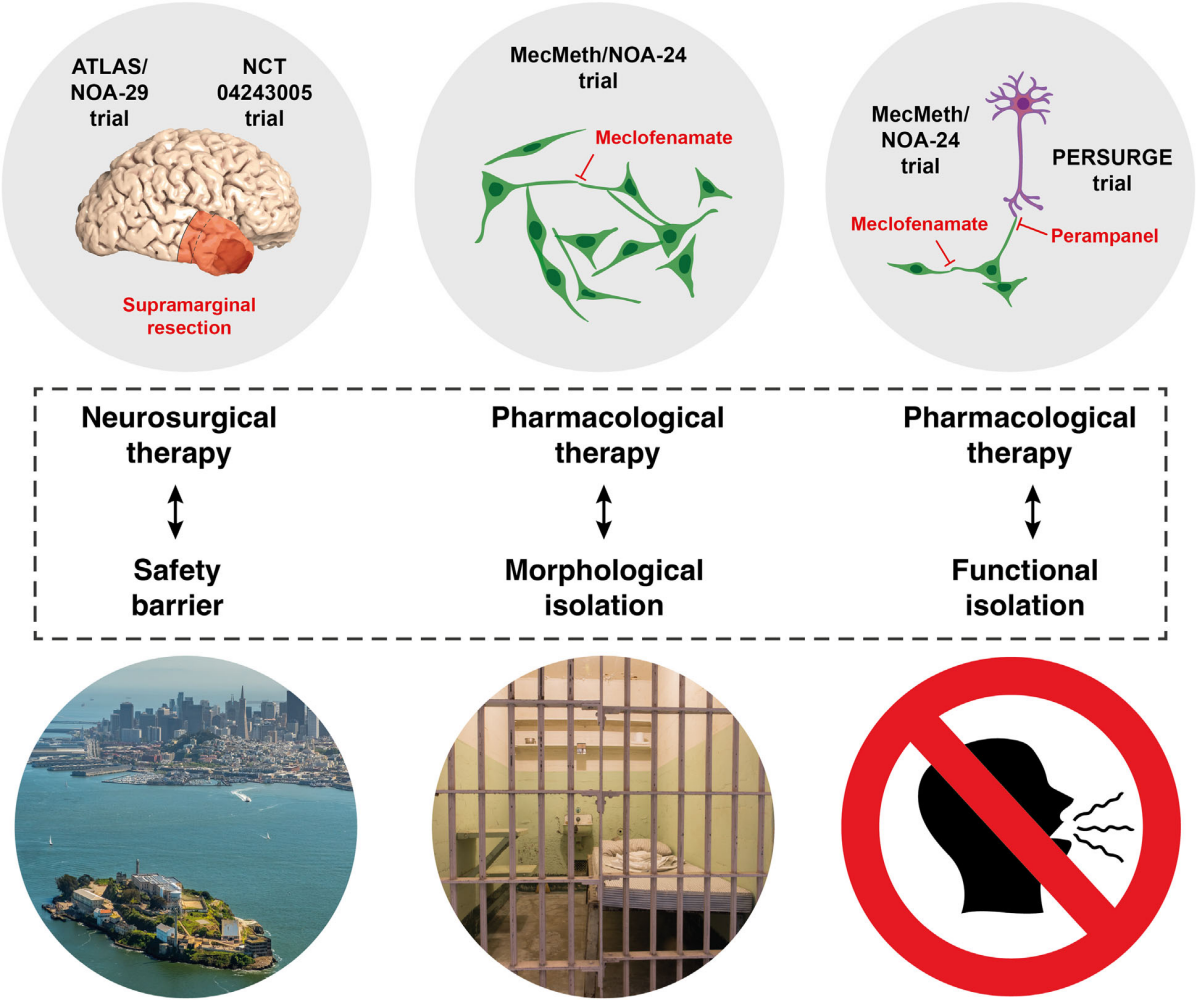
The Alcatraz‐Strategy: a translational path to disrupt malignant tumour networks in brain cancer. The Alcatraz‐Strategy draws inspiration from the former maximum‐security prison, Alcatraz, known for its success in isolating prisoners through rigorous physical and communication constraints. This strategy seeks to undermine the resilience of tumour networks in brain cancer through a comprehensive translational approach that combines advanced surgical techniques and pharmacological interventions guided by ongoing clinical trials. The strategy commences with ‘supramarginal resection’, currently undergoing evaluation in two clinical trials, ATLAS/NOA‐29 and NCT04243005. This surgical approach extends beyond the gadolinium‐enhanced regions, involving the excision of infiltrated brain tissue, which includes both the proximate and parts of the distal infiltrative network zones. Subsequent to surgery, pharmacological interventions aim to disrupt the residual postoperative network components. On the morphological front, MFA, investigated in the MecMeth/NOA‐24 trial, plays a key role by inhibiting the formation of TMs, leading to the morphological isolation of individual tumour cells. Functionally, network disconnection is achieved through two primary mechanisms: (1) Gap junction inhibition: Besides to its effects on the network architecture, MFA inhibits the cytosolic exchange between neighbouring tumour cells through gap junction blockage and also curtails the spread of ICWs within the network. (2) Inhibition of excitatory neuronal input: In the context of the PERSURGE trial, perampanel plays a central role. It acts as an antagonist of AMPARs at the postsynaptic tumour cell membrane within neurogliomal synapses, effectively isolating tumour cells from excitatory neuronal input and further compromising network function. AMPARs, α‐amino‐3‐hydroxy‐5‐methyl‐4‐isoxazolepropionic acid receptors; ICWs, intracellular calcium waves; MFA, meclofenamate; TMs, tumour microtubes.

This strategy finds an intriguing parallel in an entirely different context – the renowned former maximum‐security prison, Alcatraz, situated in San Francisco Bay. The geographical location of Alcatraz, surrounded by treacherous waters, served as an insurmountable physical barrier, preventing prisoners from escaping to the mainland. Within its walls, inmates were subject to exclusive solitary confinement, where each individual occupied ‘single cells’. Notably, even during the 1‐h daily exercise sessions, a prohibition on verbal communication was enforced. This comprehensive approach aimed to achieve complete isolation of the single prisoner, both morphologically in terms of physical separation and functionally in terms of communication constraints. Building upon this strategy, we have coined the term ‘Alcatraz‐Strategy’ (Fig. 3). The goal of this strategy is to achieve the greatest possible network inhibition, encompassing both spatial (morphological) and functional aspects, by synergizing advanced surgical techniques and pharmacological interventions.

In summary, the path towards translating tumour disconnection strategies in malignant brain tumours is progressively coming into focus. Based on current translational initiatives and initial clinical trials that aim to unravel the intricate cellular network dynamics of malignant brain tumours, we hope that the near future will provide insights into the efficacy of these novel concepts in enhancing the effectiveness of conventional treatment approaches.

## Conflict of interest

The authors declare no conflict of interest.

## Author contributions

MS and M‐AW conceived the manuscript. MS and A‐LP designed the figs MS, A‐LP, HD, HV, UH and M‐AW substantially contributed to the discussion of the content. MS, HD, HV, UH and M‐AW wrote the article. MS, A‐LP, GK‐M, PS, MDS, K‐MD, HD, HV, UH and M‐AW reviewed and/or edited the manuscript before submission.

## Ethical approval

The study methodologies conformed to the standards set by the Declaration of Helsinki. The publication of patient‐related data including the MRI‐images was approved by the local ethics committee at the University of Bonn (228/19) and written informed consent was obtained.
